# Prognosis for different patterns of distant metastases in patients with uterine cervical cancer: a population-based analysis

**DOI:** 10.7150/jca.37390

**Published:** 2020-01-14

**Authors:** Yiran Zhang, Ying Guo, Xinzi Zhou, Xuena Wang, Xiaofei Wang

**Affiliations:** 1 International Medical Center, Tianjin First Central Hospital, Tianjin, China.; 2 Nursing department, Tianjin First Central Hospital, Tianjin, China.

**Keywords:** Uterine cervical cancer, SEER, distant metastases, prognostic factor

## Abstract

**Background**: Uterine cervical cancer (UCC) is a common malignant tumor in women. We conducted this work to provide a though description on the patterns of distant metastases and to investigate the relevant factors for prognosis of UCC patients based on a large population.

**Patients and methods**: UCC patients with FIGO stage IVB, being the study group, were identified from the Surveillance, Epidemiology, and End Result (SEER) database from 2010 to 2016. UCC patients with same inclusion criteria, being validation group, were identified from Tianjin First Central Hospital from 2004 to 2017. Patterns of distant metastases were described according to the number of metastatic sites. Survival of different patterns were calculated and prognostic factors were investigated by Cox hazard regression analysis.

**Results**: Distant metastases were recorded in 1448 UCC patients among whom 295 patients (30.8%) developed metastases in two or more organs. Compared with the median OS of 8 (95%CI, 7.07-8.93) months in the patients with the single site, worse survival of 5 (95%CI, 4.29-5.71) months was noticed in the patients with multiple metastases. Age ≥ 65 years, black race, higher grade, higher T stage and more metastatic sites were proved to be the prognostic factors for all the patients in the advanced stage and the results were partly validated in the validation cohort. Moreover, black race and higher T-stage for the patients with the single metastatic sites and age ≥ 65, uninsured status, surgical treatment and metastatic pattern for the patients with two metastatic sites were prognostic factors. No independent factor was found in patients with three or more metastases.

**Conclusion**: Around 70% of the patients suffered one site distant metastasis in UCC patients with IVB stage while 30% with multiple metastases and a significantly reduced survival. Different survivals and prognostic factors were noticed for different patterns of distant metastases.

## Introduction

Uterine cervical cancer (UCC) is a common cancer in women. The incidence decreased in the developed countries in recent decades due to the HPV prevention strategy and cervical cancer screening 1. However, as previously reported, the incidence of UCC increased by 2.6% per year (1.1%, 4.2%) in Japan after 1997 2. It was also thought to be a crucial problem in other countries 1,3 and the UCC patients lost workdays annually up to 8 years after diagnosis 4.

Distant metastasis was accepted to be one of the significant characteristics in the advanced cancer. Brain metastases were reported in a series of cases after primary diagnosis of UCC 5 or as the initial presentation 6. Such distant metastases were further reported in the studies with the limited samples 7,8. Bone metastases 9,10 and lung metastases 11 were also descripted based on the relatively large population. As to the effect of distant metastases on the survival of patients, around 13% of UCC patients were diagnosed at an advanced stage, leading to the decreased survival 12. The median survival after bone metastases was reported to be only 5 months 13.

Prognostic factors in the cancer patients with distant metastases have been widely studied 14-16. In the recent study, some independent factors resulting in poor survival were found in UCC patients including the extra-skeletal metastases, performance status (level 3 to 4), radiation and/or chemotherapy, multiple bone metastases, and a bone metastases-free interval of <12 months 13. Another study, analyzing 56 UCC patients with lung metastases, revealed the prolonged overall survival (OS) in patients with less lung lesions or after surgical resection 11. These studies described the survival trend in UCC patients with different distant metastases. However, limited sample size and different data resource made the results difficult to be integrated to analyze the performance of different patterns of distant metastases.

Thus, we conducted the present study based on the Surveillance, Epidemiology, and End Results (SEER) database and our own institution. The patterns of distant metastases were described and the prognostic factors were investigated in patients based on the number and patterns of metastatic sites.

## Materials and Methods

**Figure 1:**
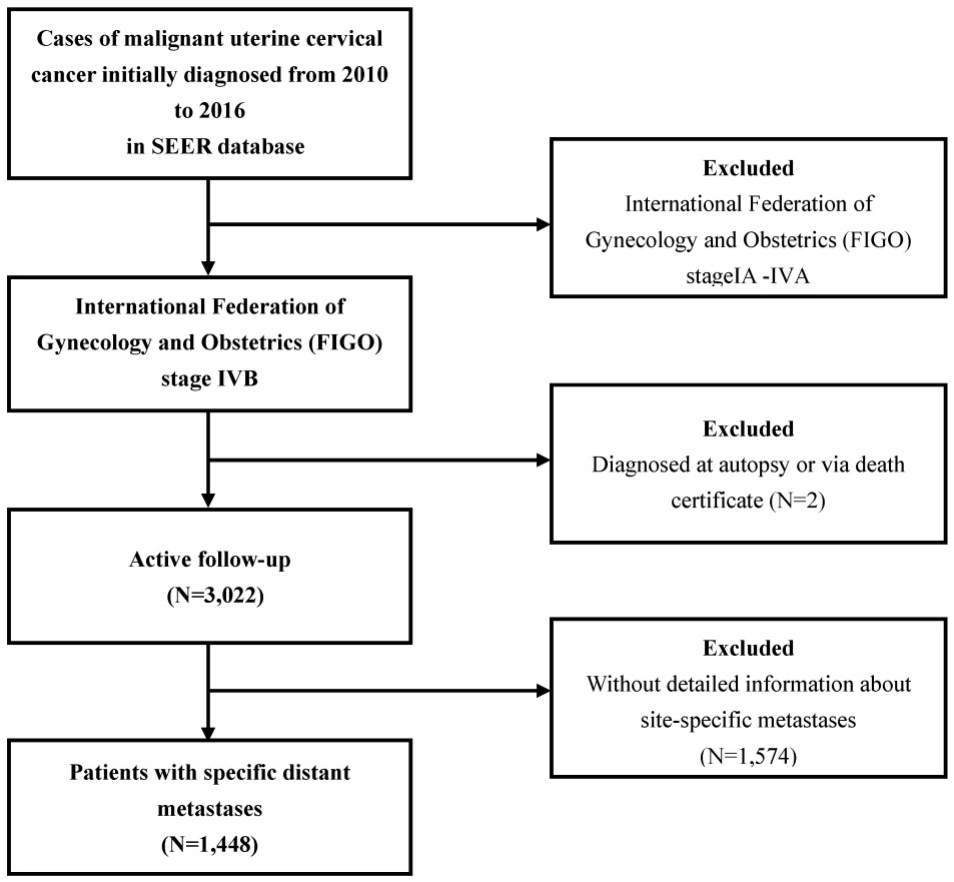
Flowchart of the patient selection in the study group.

**Figure 2:**
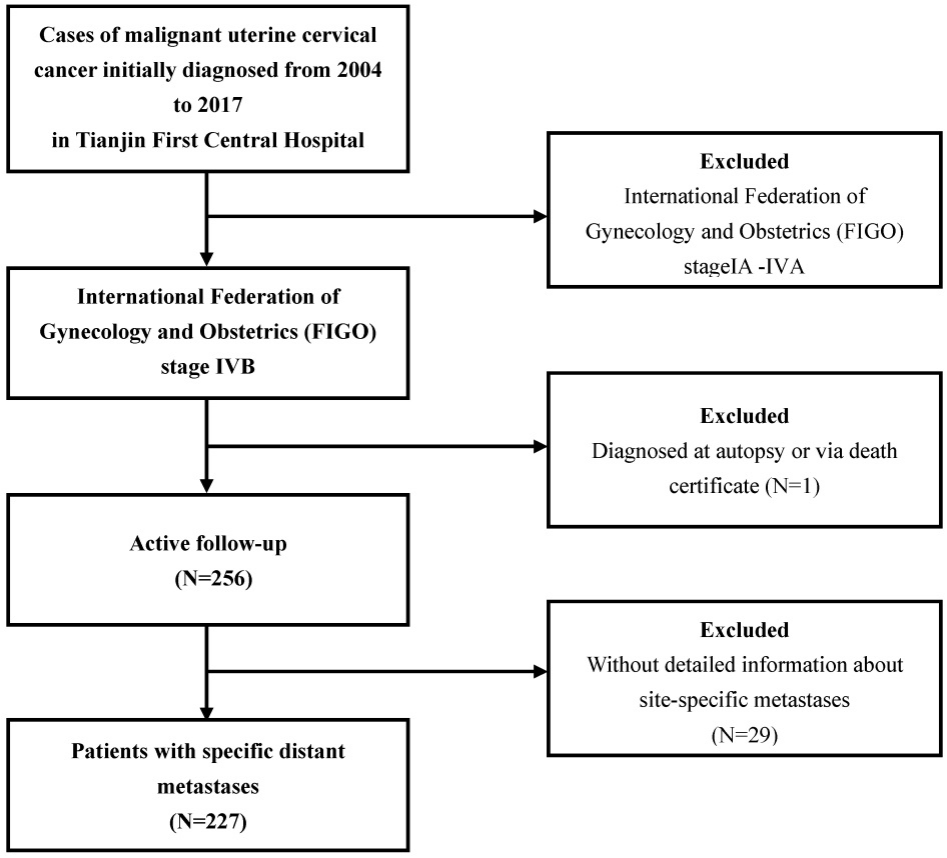
Flowchart of the patient selection in the validation group.

The patterns of distant metastases for UCC patient in both groups were described. The data of the study group were extracted from National Cancer Institute Surveillance, Epidemiology and End Results (SEER) database (https://seer.cancer.gov/), which approximately covers 28% of population in the USA. Since the details of metastases were not available before 2010, UCC patients with International Federation of Gynecology and Obstetrics (FIGO) stage IVB who were diagnosed between 2010 and 2016 in SEER database were collected. The data of the validation group were collected from Tianjin First Central Hospital, diagnosed between 2004 and 2017. The exclusion criteria were as follows: patients diagnosed at autopsy or via death certificate, without detailed information about site-specific metastases (including the bone, brain, liver, and lung). The flow-chart of the selection for study group was listed in Figure 1. After getting the patterns and the prognostic factors in study group, we validated the results based on our own institute data. The procedure for the selection of validation cohort was described as Figure 2.

### Statistical analysis

The following demographic and clinicopathological variables were collected in the study group: age, race, insurance recode, marital status, tumor grade, histological subtype (including adenocarcinoma, squamous cell carcinoma and other subtypes), T stage, N stage, number of distant metastases and surgical treatment. Aforementioned variables except race and insurance recode were collected in our validation group.

The differences in the incidence of demographic and clinicopathological variables were analyzed by the chi-squared (*χ*^2^) test. The primary outcome of the survival analysis was the overall survival (OS), which was defined from the time of UCC diagnosis to death. Survival duration was obtained using the Kaplan-Meier method, the differences between the curves were tested by Log-rank test. Cox proportional hazard regression analysis was performed for revealing the prognostic factors in UCC patients with FIGO stage IVB. Variables with *P* < 0.05 in the univariate Cox regression analysis were then further analyzed using a multivariate regression analysis.

### Ethics statement

The SEER database is a free database, and the data released by the SEER database do not require informed patient consent, because cancer is a reportable disease in every state of the USA. The present study complied with the 1964 Helsinki Declaration and its later amendments or comparable ethical standards.

## Results

### Characteristics of the patients

According to the aforementioned inclusion criteria, a total of 1448 UCC patients with FIGO stage IVB were included in the study group. The mean age was 57.53 ± 14.35 years, most patients were white race (72.4%), and unmarried (60.6%). A total of 1305 (90.1%) patients got medical insurance. After excluding 35.4% of the patients whose information on grade were unrecorded, the main grades of primary tumor were grade III - IV (44.8%). The most common histological subtype was squamous cell carcinoma (58.3%). About 51.1% of the patients were diagnosed with T3 and T4 stage, while 29.5% with T1-2 stage and 19.4% with unknown T stage. Besides, only 8.4% of the patients received surgical treatment. Baseline demographic and clinical characteristics of patients in study group were shown in Table 1.

### Sites of distant metastases

In the study group, lung was the most common site of distant metastases (n= 941, 65.0%), followed by bone (n=520, 35.9%), liver (n=466, 32.2%), and brain (n=81, 5.6%). A total of 1002 patients (69.2%) showed single distant metastatic site while 446 (30.8%) patients showed two or more distant metastatic sites. The distributions of the sites of distant metastases in study group and validation group are shown in Table 2.

### Survival outcomes in different patterns of metastases

**Figure 3:**
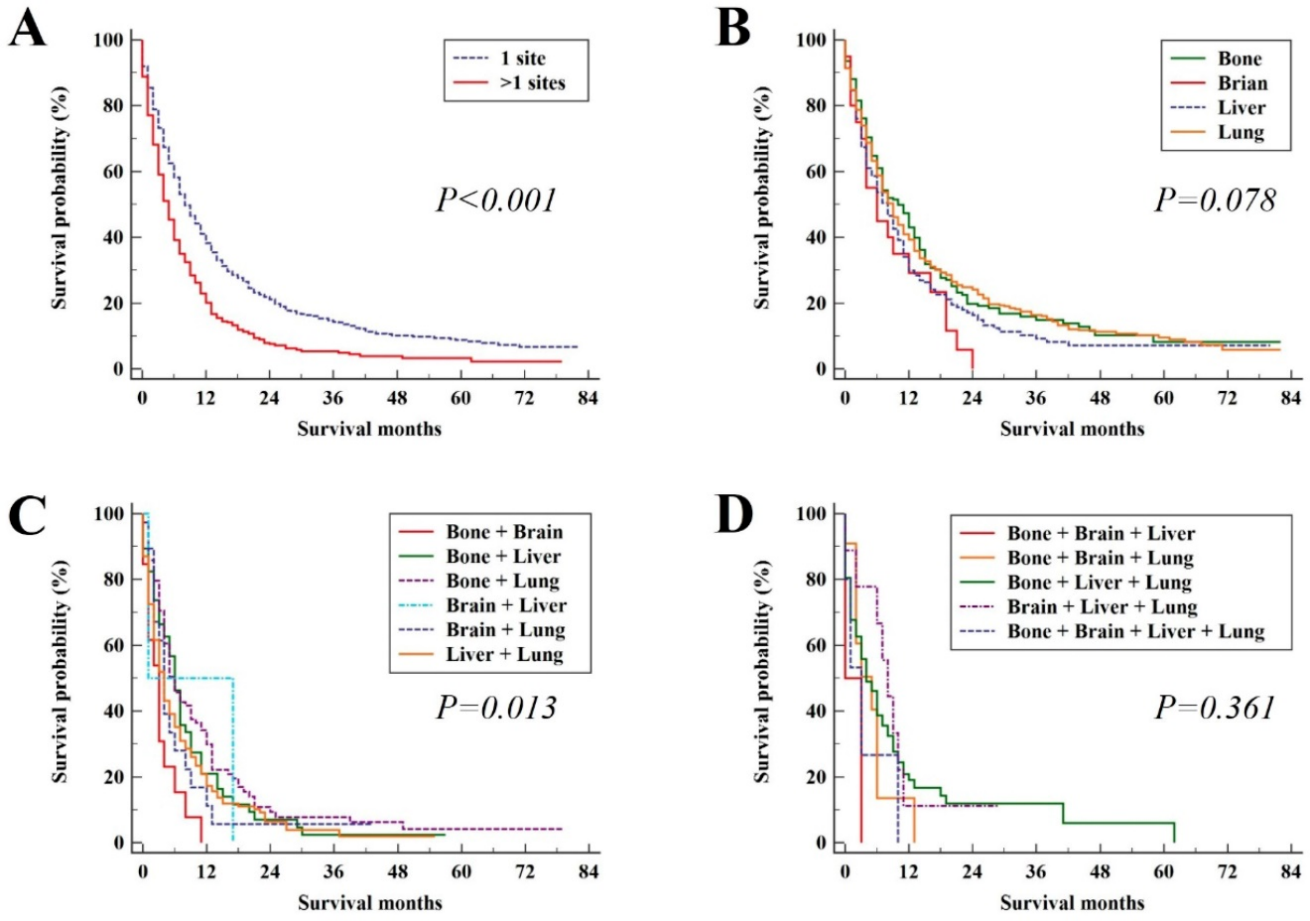
Kaplan-Meier analysis of overall survival for UCC patients in the study group (A), single site of distant metastases (B), two sites of distant metastases (C), and patients with three or more sites of distant metastases (D).

In the study group, the median survival time was 7 months (95%CI, 6.38 - 7.62). The median OS and 95% CI for patients with different metastatic patterns were shown in Table 2. The median OS for patients with the single metastatic site and multiple metastatic sites was 8 (95%CI, 7.07 - 8.93) and 5 (95%CI, 4.29 - 5.71) months, respectively (*P* < 0.001, Figure 3A). For patients with only bone, brain, liver and lung metastasis, the median OS was 10 (95%CI, 7.78 - 12.22), 6 (95%CI, 1.64 - 10.36), 8 (95%CI, 6.11 - 9.98) and 9 (95%CI, 7.85 - 10.15) months, respectively (*P* = 0.078, Figure 3B). In patients with two distant metastatic sites, there were significant difference among metastatic pattern (*P* = 0.013, Figure 3C). No significant difference among metastatic pattern was found in the patients with three or four distant metastatic sites (*P* = 0.361, Figure 3D).

### Prognostic factors for UCC patients in different patterns of metastases

#### Prognostic factors for total UCC patients

In the study group, patient with age ≥ 65 years old (HR = 1.35; 95% CI, 1.03 -1.77; *P* = 0.031), black race (HR = 1.27; 95% CI, 1.03 -1.56; *P* = 0.028), higher grade (HR = 1.28; 95% CI, 1.08 - 1.53; *P* = 0.006), higher T-stage (HR = 1.27; 95% CI, 1.08 - 1.51; *P* = 0.005), and two or more metastatic sites were significantly associated with OS in the Cox regression analysis. The HR and 95%CI for patients with two or more than two metastatic sites were 1.65 (95% CI, 1.37 - 1.99; *P* < 0.001) and 1.68 (95% CI, 1.19 - 2.39; *P* = 0.004), respectively. Trend chi-square test confirmed the worse OS in patients with more than two metastatic sites compared with those with two metastatic sites.

However, the factor of two metastatic sites was the only independent prognostic factor for all patients in the validation group. The aforementioned prognostic factors were shown in Table 3.

#### Patients with single distant metastatic site

As shown in Table 4, for patients with one metastatic site, univariate Cox regression analysis showed that the following factors were significantly associated with overall survival: patient with age ≥ 65 (HR = 1.61; 95% CI, 1.25 - 2.07; *P* < 0.001), black race (HR = 1.28; 95% CI, 1.07 - 1.53; *P* = 0.006), married status (HR = 0.81; 95% CI, 0.69 - 0.94; *P* = 0.007), higher grade (HR = 1.23; 95% CI, 1.02 - 1.49; *P* = 0.029), higher T-stage (HR = 1.44; 95% CI, 1.21 - 1.70; *P* < 0.001), surgical treatment (HR = 0.60; 95% CI, 0.45 - 0.79; *P* < 0.001). Although the variable of metastatic site was not significant in univariate analysis, it was included in further multivariate analysis. The multivariate analysis identified the following variables as independent factors for prognosis: black race and higher T-stage. Metastatic site was not the independent prognostic factor in patients with the single distant metastatic site. In the validation group, N-stage was the only independent prognostic factor for patients with one site metastasis. The aforementioned prognostic factors were shown in Table 4.

#### Patients with two or more distant metastatic sites

As shown in Table 5, univariate Cox regression analysis suggested that the following factors being significantly associated with the survival of patients who developed two distant metastatic sites: age, race, insurance, metastatic pattern and surgical treatment. The multivariate analysis suggested that age ≥ 65, uninsured status, surgical treatment and metastatic pattern were the independent prognostic factors in patients with two distant metastatic sites. In the validation group, N-stage was the only factor associated with overall survival in patients with two distant metastatic sites.

For patients with three or four distant metastatic sites in study group, no factors were significantly associated with overall survival and metastatic site was not the independent prognostic factor in patients with three or more distant metastatic sites. In the validation group, marital status was the only factor associated with overall survival. The results of Univariate Cox regression analysis for patients with three or more distant metastatic sites in both study group and validation group were shown in Table 6.

#### Validation of the survival and for prognostic factors in the validation group

In the validation group, from 2004 to 2017 in Tianjin First Central Hospital, 227 UCC patients were included. The baseline demographic and clinical characteristics for patients in validation group were shown in Table 1.

In our validation cohort, lung was also the most common site of distant metastases (n=152, 67.0%), followed by bone (n=94, 41.4%), liver (n=68, 30.0%), and brain (n=14, 6.2%). A total of 151 (66.5%) patients showed single distant metastatic site while 76 (33.5%) patients showed two or three distant metastatic sites. No patient showed both lung, bone, liver and brain metastasis at the same time. The distribution of the distant metastatic sites for two groups was listed in Table 2.

**Figure 4:**
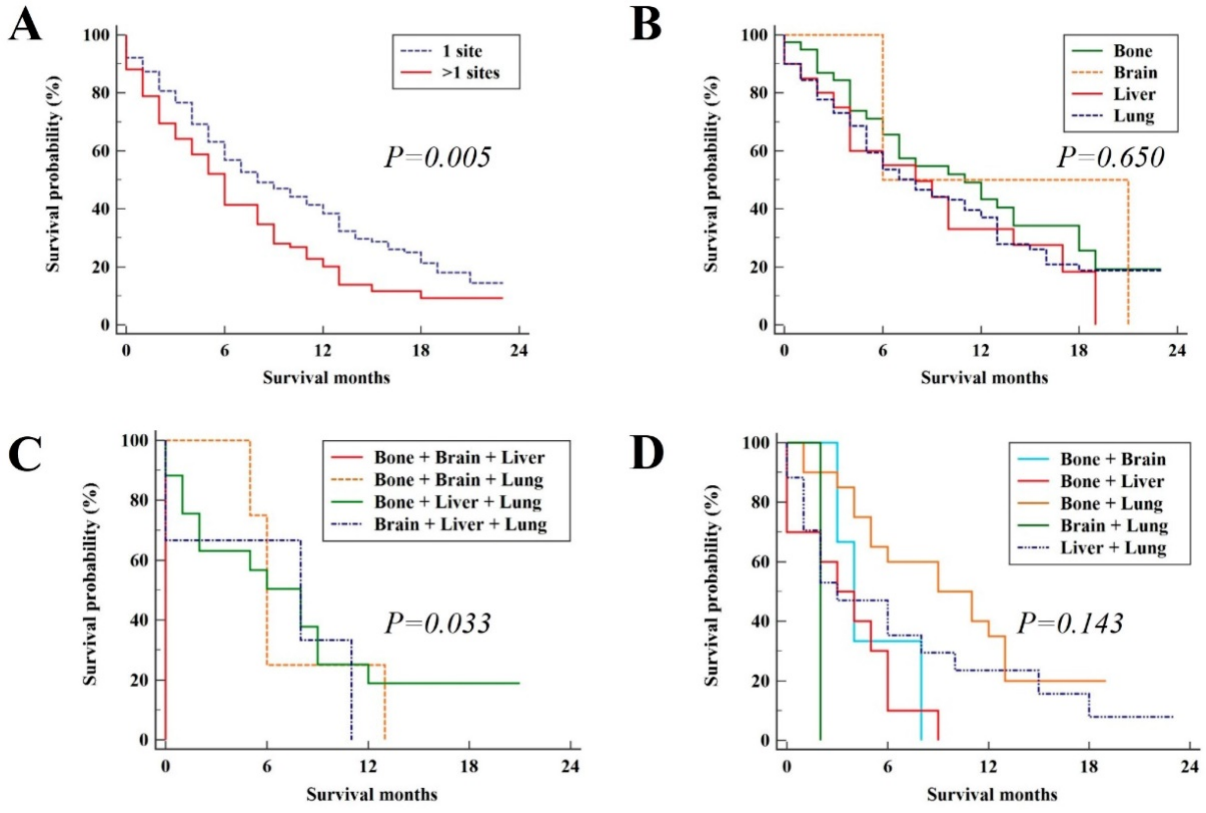
Kaplan-Meier analysis of overall survival for UCC patients in the validation group (A), single site of distant metastases (B), two sites of distant metastases (C), and patients with three or more sites of distant metastases (D).

In the validation group, the median survival time was 7 months (95%CI, 5.70 - 8.30). The median OS for patients with the single metastatic site and multiple metastatic sites was 8 (95%CI, 5.43 - 10.57) and 6 (95%CI, 4.72 - 7.28), respectively with significant difference (*P* = 0.005, Figure 4A). The survival outcome of validation group was similar to the results in the study group. For patients with single metastatic site and patients with three distant metastatic sites, there was no significant difference among metastatic pattern (P = 0.650, Figure 4B and *P* = 0.143, Figure 4D, respectively). While for patients with two distant metastatic sites, there were significant differences among metastatic pattern (*P* = 0.033, Figure 4C).

## Discussion

In the present study with the large population, to our knowledge, it was the first time to perform a thorough investigation on both the survival and prognostic factors for different patterns of distant metastases in initial UCC patients at the FIGO stage ⅣB. Around 30% of all the patients suffered multiple distant metastases at the end of their lives. Higher incidence of multiple metastases was reported in another study 8. The present study revealed that involvement of extra metastases significantly reduced patient's survival. Meanwhile, different prognostic factors were found in patients with various metastatic patterns.

Most of the patients suffered single distant metastasis among which lung was the most common site in the present study, followed by bone, liver, and brain. A similar metastatic distribution was reported in another study including metastases to lung in 11 patients, bone in 7 patients and liver in 4 patients 17. However, a much larger sample size in this study provided us a chance to evaluate the diversities in metastatic site-specific survival. Cox analysis found no significant difference in patients with any one metastasis in this study. UCC patients with only bone metastasis showed a trend of better survival than the patients with other three types of distant metastases. Comparable median OS of 8 months in patients with lung metastasis was confirmed in both previous and the present study. Comparable median survival of 10 months 9 and worse survival (5 months) 13 after diagnosis of bone metastasis were shown in other studies. Brain metastasis in UCC patients showed the worst median OS of only 4 months which was consistent with the reported result 8. Besides, the multivariate analysis identified the higher grade and higher T-stage as prognostic factors for UCC patients with the single site of distant metastases.

With the exception of the single site of metastasis, 295 of 979 patients suffered metastases in two or more organs. Different configurations of two metastatic sites showed different patients' survivals. Compared with the patient with metastases to bone plus brain, better survivals were noticed in patients with metastases to bone plus liver, bone plus lung, and liver plus lung. No significant difference was noticed between patients with brain plus bone, liver or lung. Therefore, more attention should be paid for the screening of brain metastases even though the relevant incidence was low. As to the factor's analyses, only the uninsured status was found to be the independent prognostic factor. For patients with three or more metastatic sites, no significant difference was found among metastatic patterns. The late stage without any effective treatments may be the main reason why no difference or prognostic factors were found. Timely screening and proper treatment should be performed in order to slow down the progress into multiple metastases and to improve the survival.

Treatment for patients with distant metastases was accepted to be the important prognostic factor and was investigated in the previous studies. A study reported a significantly longer 5-year survival in patients after definitive radiotherapy and chemoradiotherapy 18. Surgical resections of metastatic pulmonary lesions were performed in 12 patients with longer survival than those who did not receive the surgery 11. A review concluded that multimodal therapy can guarantee a better survival 12. Only 7.9% of the UCC patients received surgery of primary site in our study and no significant impact of surgery on the survival was found. The records on the surgery on the metastatic lesion in the SEER database were not available. Besides, no information of radiotherapy or chemotherapy can be pooled, making it impossible to evaluate the influence on survival. All the treatments should be further investigated based on larger population.

Although the present study identified the largest cohort of single and multiple distant metastases in UCC patients with FIGO stage ⅣB, the limitations should be revealed. In this study, we only analyzed the distant metastases to organs of lung, bone, liver and brain. The metastases to distant lymph nodes and peritoneal spread were not investigated because of no available data in the SEER database. The sequence of each metastasis in patients with multiple sites may be the important confusing factor to survival and should be further investigated in the larger population with related information. Limited number of patients in some patterns of metastases may result in bias in survival calculation and comparation. Moreover, some important factors including human papillomavirus (HPV) infection and special gene expression should be investigated in future study. Moreover, due to the limited sample size in the validation group, not all the prognostic factors were validated, and more studies with larger sample size were needed in future.

In summary, the lung, bone, liver, and brain were the common metastatic sites in patients with UCC at FIGO stage ⅣB, among whom 30% patients suffered multiple metastases. Decreased survival was confirmed in patients with increased number of metastatic sites. Higher grade, higher T stage, black race, unmarried status and more sites were risk factors in all the patients with distant metastases. Different performance was showed in survival and prognostic factors among the patterns of distant metastases.

## Figures and Tables

**Table 1:** Description of the SEER study population and validation group with uterine cervical cancer by distant metastases at diagnosis.

Subject characteristics	Study group				Validation group			
	Bone metastases	Brain metastases	Liver metastases	Lung metastases	Bone metastases	Brain metastases	Liver metastases	Lung metastases
	Yes	Yes	Yes	Yes	Yes	Yes	Yes	Yes
	(N=520, %)	(N=81, %)	(N=466, %)	(N=941, %)	(N=94, %)	(N=14, %)	(N=68, %)	(N=152, %)
**Age**								
≤ 40	72 (40.7)	12 (6.8)	67 (37.9)	103 (58.2)	8 (34.8)	2 (8.7)	4 (17.4)	18 (78.3)
41-64	307 (37.2)	54 (6.5)	260 (31.5)	535 (64.8)	65 (49.2)	9 (6.8)	47 (35.6)	85 (64.4)
≥ 65	141 (31.7)	15 (3.4)	139 (31.2)	303 (68.1)	21 (29.2)	3 (4.2)	17 (23.6)	49 (68.1)
**Race**								
White	384 (36.6)	61 (5.8)	318 (30.3)	685 (65.3)	NA	NA	NA	NA
Black	87 (32.7)	12 (4.5)	100 (37.6)	164 (61.7)	NA	NA	NA	NA
Others	49 (37.1)	7 (5.3)	48 (36.4)	92 (69.7)	NA	NA	NA	NA
Unknown	0 (0)	1 (100.0)	0 (0)	0 (0)	NA	NA	NA	NA
**Insurance Record**								
Uninsured	38 (33.3)	8 (7.0)	42 (36.8)	62 (54.4)	NA	NA	NA	NA
Insured	474 (36.3)	72 (5.5)	416 (31.9)	858 (65.7)	NA	NA	NA	NA
Unknown	8 (27.6)	1 (3.4)	8 (27.6)	21 (72.4)	NA	NA	NA	NA
**Marital status**								
Unmarried	307 (35.0)	49 (5.6)	271 (30.9)	574 (65.4)	53 (40.5)	8 (6.1)	43 (32.8)	84 (64.1)
Married	191 (37.9)	26 (5.2)	165 (32.7)	322 (63.9)	38 (44.2)	4 (4.7)	22 (25.6)	58 (67.4)
Unknown	22 (33.3)	6 (9.1)	30 (45.5)	45 (68.2)	3 (30.0)	2 (20.0)	3 (30.0)	10 (100.0)
**Grade**								
Grade I-II	94 (32.8)	13 (4.5)	67 (23.3)	184 (64.1)	18 (38.3)	3 (6.4)	10 (21.3)	33 (70.2)
Grade III-IV	241 (37.1)	47 (7.2)	209 (32.2)	438 (67.5)	42 (38.9)	9 (8.3)	35 (32.4)	73 (67.6)
Unknown	185 (36.1)	21 (4.1)	190 (37.1)	319 (62.3)	34 (47.2)	2 (2.8)	23 (31.9)	46 (63.9)
**Histology**								
AC	87 (36.0)	11 (4.5)	82 (33.9)	157 (64.9)	15 (41.7)	4 (11.1)	11 (30.6)	26 (72.2)
SCC	301 (35.7)	51 (6.0)	230 (27.3)	566 (67.1)	52 (39.7)	7 (5.3)	32 (24.4)	89 (67.9)
Others	132 (36.5)	19 (5.2)	154 (42.5)	218 (60.2)	27 (45.0)	3 (5.0)	25 (41.7)	37 (61.7)
**T stage**								
T1-2	155 (36.3)	24 (5.6)	147 (34.4)	267 (62.5)	30 (40.0)	5 (6.7)	23 (30.7)	53 (70.7)
T3-4	267 (36.1)	28 (3.8)	221 (29.9)	482 (65.1)	49 (44.1)	4 (3.6)	32 (28.8)	68 (61.3)
Unknown	98 (34.9)	29 (10.3)	98 (34.9)	192 (68.3)	15 (36.6)	5 (12.2)	13 (31.7)	31 (75.6)
**N stage**								
N0	133 (32.0)	16 (3.8)	140 (33.7)	260 (62.5)	24 (33.3)	1 (1.4)	23 (31.9)	45 (62.5)
N1	325 (39.3)	46 (5.6)	250 (30.2)	539 (65.1)	61 (47.3)	11 (8.5)	36 (27.9)	87 (67.4)
Unknown	62 (30.4)	19 (9.3)	76 (37.3)	142 (69.6)	9 (34.6)	2 (7.7)	9 (34.6)	20 (76.9)
**Surgical treatment**								
No Surgery	486 (36.7)	73 (5.5)	424 (32.0)	866 (65.3)	86 (42.4)	13 (6.4)	63 (31.0)	136 (67.0)
Surgery	34 (28.1)	8 (6.6)	42 (34.7)	74 (61.2)	8 (33.3)	1 (4.2)	5 (20.8)	16 (66.7)
Unknown	0 (0)	0 (0)	0 (0)	1 (100.0)	0 (0)	0 (0)	0 (0)	0 (0)

Abbreviations: SEER = Surveillance, Epidemiology, and End Result; AC = adenocarcinoma; SCC = squamous cell carcinoma.

**Table 2:** Patterns of distant metastases and overall survival time for uterine cervical cancer patients in the study and the validation group.

Sites of distant metastases	Study group		Validation group	
	N (%)	Median OS (95%CI)	N (%)	Median OS (95%CI)
One site of distant metastases				
Bone	236 (16.3)	10 (7.78 - 12.22)	39 (17.2)	11 (5.30 - 16.70)
Brain	20 (1.4)	6 (1.64 - 10.36)	2 (0.9)	6 (-)
Liver	177 (12.2)	8 (6.11 - 9.89)	20 (8.8)	8 (1.97 - 14.04)
Lung	569 (39.3)	9 (7.85 - 10.15)	90 (39.6)	8 (5.24 - 10.76)
Two sites of distant metastases				
Bone + Brain	13 (0.9)	3 (1.37 - 4.63)	3 (1.3)	4 (2.40 - 5.60)
Bone + Liver	57 (3.9)	6 (4.71 - 7.29)	10 (4.4)	3 (0 - 6.10)
Bone + Lung	114 (7.9)	6 (4.43 - 7.57)	20 (8.8)	9 (3.52 - 14.48)
Brain + Liver	2 (0.1)	1	0	-
Brain + Lung	19 (1.3)	4 (2.99 - 5.01)	1 (0.4)	2 (-)
Liver + Lung	132 (9.1)	4 (3.06 - 4.94)	17 (7.5)	3 (0 - 6.36)
Three sites of distant metastases				
Bone + Brain + Liver	2 (0.1)	-	1 (0.4)	0 (-)
Bone + Brain + Lung	11 (0.8)	5 (0.48 - 9.52)	4 (1.8)	6 (5.15 - 6.85)
Bone + Liver + Lung	82 (5.7)	4 (2.04 - 5.96)	17 (7.5)	8 (4.22 -11.78)
Brain + Liver + Lung	9 (0.6)	8 (5.08 - 10.92)	3 (1.3)	8 (0 - 20.80)
Four sites of distant metastases				
Bone + Brain + Liver + Lung	5 (0.3)	3 (0.51 - 5.49)	0	-

Abbreviations: FIGO=International Federation of Gynecology and Obstetrics, OS=Overall Survival

**Table 3:** Univariate and Multivariate Cox regression analysis of prognostic factors for all FIGO stage IVB UCC patients in study and validation group.

Variable	Study group						Validation group					
	Univariate analysis			Multivariate analysis			Univariate analysis			Multivariate analysis		
	HR	95%CI	*P-value*	HR	95%CI	*P-value*	HR	95%CI	*P-value*	HR	95%CI	*P-value*
**Age**												
≤ 40	1			1			1			-		
41-64	1.05	0.87 - 1.27	*0.614*	0.97	0.75 - 1.25	*0.794*	0.75	0.46 - 1.23	*0.256*	-	-	-
≥ 65	1.51	1.23 - 1.84	*<0.001*	1.35	1.03 - 1.77	*0.031*	1.42	0.85 - 2.37	*0.185*	-	-	-
**Race**												
White	1			1			NA					
Black	1.25	1.07 - 1.44	*0.004*	1.27	1.03 - 1.56	*0.028*	NA	NA	*NA*	NA	NA	*NA*
Others	0.87	0.70 - 1.07	*0.177*	0.97	0.72 - 1.30	*0.813*	NA	NA	*NA*	NA	NA	*NA*
**Insurance Recode**												
Uninsured	1			-			NA			NA		
Insured	0.87	0.70 - 1.08	*0.201*	-	-	-	NA	NA	*NA*	NA	NA	*NA*
**Marital status**												
Unmarried	1			1			1			-		
Married	0.86	0.76 - 0.97	*0.014*	0.89	0.75 - 1.05	*0.160*	0.93	0.68 - 1.27	*0.635*	-	-	-
**Grade**												
Grade I- II	1			1			1			-		
Grade III- IV	1.31	1.12 - 1.53	*0.001*	1.28	1.08 - 1.53	*0.006*	0.951	0.65 - 1.39	*0.796*	-	-	-
**Histology**												
AC	1			-			1			-		
SCC	1.01	0.86 - 1.18	*0.945*	-	-	-	0.694	0.46 - 1.05	*0.081*	-	-	-
Others	1.17	0.98 - 1.41	*0.087*	-	-	-	0.966	0.61 - 1.52	*0.880*	-	-	-
**T stage**												
T1-2	1			1			1			-		
T3-4	1.29	1.12 - 1.47	*<0.001*	1.27	1.08 - 1.51	*0.005*	0.903	0.65 - 1.26	*0.552*	-	-	-
**N stage**												
N0	1			-			1			-		
N1	1.00	0.88 - 1.15	*0.984*	-	-	-	0.892	0.64 - 1.24	*0.496*	-	-	-
**Number of metastasis sites**												
1	1			1			1			1		
2	1.56	1.36 - 1.79	*<0.001*	1.65	1.37 - 1.99	*<0.001*	1.508	1.07 - 2.13	*0.021*	1.48	1.05 - 2.10	*0.026*
≥ 3	1.70	1.36 - 2.13	*<0.001*	1.68	1.19 - 2.39	*0.004*	1.542	0.96 - 2.47	*0.070*	1.46	0.91 - 2.34	*0.119*
**Surgical treatment**												
No Surgery	1			1			1			1		
Surgery	0.63	0.50 - 0.79	*<0.001*	0.76	0.58 - 1.00	*0.053*	0.592	0.35 - 1.01	*0.053*	0.62	0.37 - 1.06	*0.083*

**Table 4:** Univariate and Multivariate Cox regression analysis of prognostic factors for FIGO stage IVB UCC patients with single distant metastatic site in the study group and the validation group.

Variable	Study group						Validation group					
	Univariate analysis			Multivariate analysis			Univariate analysis			Multivariate analysis		
	HR	95%CI	*P-value*	HR	95%CI	*P-value*	HR	95%CI	*P-value*	HR	95%CI	*P-value*
**Age**												
≤ 40	1			1			1			-		
41-64	1.00	0.79 - 1.28	*0.972*	0.90	0.65 - 1.25	*0.538*	0.67	0.36 - 1.26	*0.212*	-	-	-
≥ 65	1.61	1.25 - 2.07	*<0.001*	1.38	0.98 - 1.94	*0.062*	1.41	0.75 - 2.67	*0.286*	-	-	-
**Race**												
White	1			1			NA					
Black	1.28	1.07 - 1.53	*0.006*	1.37	1.07 - 1.75	*0.012*	NA	NA	*NA*	NA	NA	*NA*
Others	0.83	0.62 - 1.10	*0.186*	0.96	0.64 - 1.44	*0.835*	NA	NA	*NA*	NA	NA	*NA*
**Insurance Recode**												
Uninsured	1			-			NA			NA		
Insured	0.93	0.72 - 1.21	*0.595*	-	-	-	NA	NA	*NA*	NA	NA	*NA*
**Marital status**												
Unmarried	1			1			1			-		
Married	0.81	0.69 - 0.94	*0.007*	0.90	0.73 - 1.11	*0.318*	0.74	0.50 - 1.10	*0.133*	-	-	-
**Grade**												
Grade I- II	1			1			1			-		
Grade III- IV	1.23	1.02 - 1.49	*0.029*	1.22	0.99 - 1.51	*0.057*	0.88	0.55 - 1.41	*0.596*	-	-	-
**Histology**												
AC	1			-			1			-		
SCC	0.95	0.78 - 1.15	*0.573*	-	-	-	0.71	0.40 - 1.25	*0.232*	-	-	-
Others	1.05	0.84 - 1.32	*0.661*	-	-	-	0.87	0.46 - 1.62	*0.650*	-	-	-
**T stage**												
T1-2	1			1			1			-		
T3-4	1.44	1.21 - 1.70	*<0.001*	1.43	1.16 - 1.77	*0.001*	1.03	0.67 - 1.58	*0.904*	-	-	-
**N stage**												
N0	1			-			1			1		
N1	0.96	0.82 - 1.13	*0.622*	-	-	-	0.67	0.45 - 0.99	*0.046*	0.65	0.43 - 0.99	*0.043*
**Metastatic sites**												
Bone	1			1			1			1		
Brain	1.54	0.96 - 2.48	*0.073*	1.66	0.91 - 3.03	*0.102*	1.01	0.24 - 4.29	*0.992*	0.95	0.22 - 4.10	*0.945*
Liver	1.22	0.98 - 1.52	*0.083*	1.00	0.73 - 1.38	*0.987*	1.39	0.75 - 2.58	*0.301*	1.29	0.67 - 2.49	*0.443*
Lung	1.03	0.86 - 1.23	*0.749*	0.91	0.71 - 1.15	*0.415*	1.27	0.81 - 1.99	*0.289*	1.27	0.78 - 2.07	*0.330*
**Surgical treatment**												
No Surgery	1			1			1			1		
Surgery	0.60	0.45 - 0.79	*<0.001*	0.78	0.56 - 1.09	*0.145*	0.52	0.27 - 1.00	*0.051*	0.51	0.26 - 1.03	*0.059*

**Table 5:** Univariate and Multivariate Cox regression analysis of prognostic factors for FIGO stage ⅣB UCC patients with two sites of distant metastases in the study and the validation group.

Variable	Study group						Validation group		
	Univariate analysis			Multivariate analysis			Univariate analysis		
	HR	95%CI	*P-value*	HR	95%CI	*P-value*	HR	95%CI	*P-value*
**Age**									
≤ 40	1			1			1		
41-64	1.23	0.86 - 1.74	*0.255*	0.99	0.69 - 1.44	*0.970*	0.99	0.38 - 2.58	*0.984*
≥ 65	1.79	1.21 - 2.65	*0.004*	1.72	1.14 - 2.59	*0.010*	2.60	0.89 - 7.60	*0.080*
**Race**									
White	1			1			NA		
Black	1.39	1.04 - 1.85	*0.027*	1.237	0.92 - 1.67	*0.165*	NA	NA	*NA*
Others	0.78	0.54 - 1.13	*0.195*	0.775	0.53 - 1.14	*0.191*	NA	NA	*NA*
**Insurance Recode**									
Uninsured	1			1			NA		
Insured	0.51	0.33 - 0.79	*0.002*	0.522	0.34 - 0.81	*0.004*	NA	NA	*NA*
**Marital status**									
Unmarried	1			-			1		
Married	0.83	0.65 - 1.05	*0.117*	-	-	-	1.18	0.63 - 2.20	*0.603*
**Grade**									
Grade I- II	1			-			1		
Grade III- IV	1.35	0.97 - 1.87	*0.071*	-	-	-	1.09	0.52 - 2.30	*0.815*
**Histology**									
AC	1			-			1		
SCC	1.27	0.90 - 1.78	*0.174*	-	-	-	0.81	0.40 - 1.63	*0.550*
Others	1.40	0.97 - 2.03	*0.074*	-	-	-	0.98	0.45 - 2.16	*0.959*
**T stage**									
T1-2	1			-			1		
T3-4	1.05	0.80 - 1.37	*0.740*	-	-	-	0.87	0.45 - 1.69	*0.675*
**N stage**									
N0	1			-			1		
N1	1.12	0.85 - 1.47	*0.420*	-	-	-	2.63	1.13 - 6.11	*0.025*
**Metastatic sites**									
Bone + Brain	1			1			1		
Bone + Liver	0.47	0.25 - 0.86	*0.015*	0.44	0.24 - 0.83	*0.011*	1.27	0.35 - 4.62	*0.721*
Bone + Lung	0.40	0.22 - 0.72	*0.002*	0.36	0.20 - 0.65	*0.001*	0.40	0.11 - 1.42	*0.158*
Brain + Liver	0.46	0.10- 2.06	*0.312*	0.99	0.21 - 4.63	*0.984*	NA	NA	*NA*
Brain + Lung	0.57	0.28 - 1.18	*0.133*	0.56	0.27 - 1.17	*0.123*	2.14	0.22 - 21.19	*0.517*
Liver + Lung	0.56	0.31 - 0.99	*0.047*	0.50	0.28 - 0.89	*0.018*	0.64	0.18 - 2.27	*0.485*
**Surgical treatment**									
No Surgery	1			1			1		
Surgery	0.64	0.42 - 0.99	*0.046*	0.509	0.32 - 0.81	*0.005*	0.74	0.26 - 2.07	*0.560*

**Table 6:** Univariate Cox regression analysis of prognostic factors for FIGO stage IVB UCC patients with three or four sites of distant metastases in the study and the validation group.

Variable	Study group			Validation group		
	HR	95%CI	*P-value*	HR	95%CI	*P-value*
**Age**						
≤ 40	1			1		
41-64	1.23	0.62 - 2.43	*0.551*	0.51	0.11 - 2.28	*0.375*
≥ 65	0.91	0.43 - 1.93	*0.814*	0.74	0.12 - 4.47	*0.738*
**Race**						
White	1			NA		
Black	0.66	0.33 - 1.33	*0.249*	NA	NA	*NA*
Others	0.77	0.40 - 1.51	*0.450*	NA	NA	*NA*
**Insurance Recode**						
Uninsured	1			NA		
Insured	0.94	0.38 - 2.33	*0.897*	NA	NA	*NA*
**Marital status**						
Unmarried	1			1		
Married	1.21	0.76 - 1.94	*0.429*	4.00	1.36 - 11.8	*0.012*
**Grade**						
Grade I- II	1			1		
Grade III- IV	0.84	0.33 - 2.16	*0.717*	0.76	0.20 - 2.93	*0.691*
**Histology**						
AC	1			1		
SCC	1.00	0.55 - 1.80	*0.990*	0.85	0.18 - 3.96	*0.840*
Others	1.46	0.77 - 2.75	*0.249*	2.76	0.57 - 13.37	*0.206*
**T stage**						
T1-2	1			1		
T3-4	0.87	0.53 - 1.42	*0.576*	0.71	0.25 - 2.01	*0.521*
**N stage**						
N0	1			1		
N1	0.64	0.37 - 1.13	*0.123*	0.38	0.12 - 1.16	*0.088*
**Metastatic sites**						
Bone + Brain + Liver	1			1		
Bone + Brain + Lung	0.44	0.09 - 2.05	*0.294*	0.13	0.01 - 1.49	*0.101*
Bone + Liver + Lung	0.34	0.08 - 1.43	*0.143*	0.12	0.01 - 1.17	*0.068*
Brain + Liver + Lung	0.28	0.06 - 1.37	*0.116*	0.16	0.01 - 1.87	*0.142*
Bone + Brain + Liver + Lung	0.54	0.10 - 2.99	*0.483*	NA	NA	*NA*
**Surgical treatment**						
No Surgery	1			1		
Surgery	1.35	0.55 - 3.36	*0.513*	8.01	0.83 - 76.94	*0.072*
